# The Palaeobiology of Two Crown Group Cnidarians: *Haootia quadriformis* and *Mamsetia manunis* gen. et sp. nov. from the Ediacaran of Newfoundland, Canada

**DOI:** 10.3390/life14091096

**Published:** 2024-08-30

**Authors:** D. McIlroy, G. Pasinetti, D. Pérez-Pinedo, C. McKean, S. C. Dufour, J. J. Matthews, L. R. Menon, R. Nicholls, R. S. Taylor

**Affiliations:** 1 Department of Earth Sciences, Memorial University of Newfoundland, St. John’s, NL A1C 5S7, Canada; gpasinetti@mun.ca (G.P.); dperezpinedo@mun.ca (D.P.-P.); cmckean@mun.ca (C.M.); rodt@mun.ca (R.S.T.); 2 Department of Biology, Memorial University of Newfoundland, St. John’s, NL A1C 5S7, Canada; sdufour@mun.ca; 3 Museum of Natural History, University of Oxford, Oxford OX3 7DQ, UK; jack.matthews@oum.ox.ac.uk; 4 Department of Earth Sciences, University of Oxford, Oxford OX1 3AN, UK; menon891@btinternet.com; 5 BobNichollsArt, Bristol BS161QY, UK; bobnichollsart@gmail.com

**Keywords:** Ediacaran, Cnidaria, fossil, taphonomy, biostratinomy, Staurozoa, proterozoic

## Abstract

The Ediacaran of eastern Newfoundland preserves the world’s oldest known eumetazoan body fossils, as well as the earliest known record of fossilized muscular tissue. Re-examination of the holotype of the eight-armed *Haootia quadriformis* in terms of its morphology, the arrangement of its muscle filament bundles, and hitherto undescribed aspects of its anatomy support its interpretation as a crown staurozoan. We also document several new fossils preserving muscle tissue with a different muscular architecture to *Haootia*, but with only four arms. This new material allows us to describe a new crown group staurozoan, *Mamsetia manunis* gen. et sp. nov. This work confirms the presence of crown group medusozoan cnidarians of the Staurozoa in the Ediacaran of Newfoundland circa 565 Ma.

## 1. Introduction

During the Ediacaran period, Newfoundland lay on the eastern margin of Gondwana and was part of the Avalonian Terrane [1,2,3]. The most significant finds of Ediacaran fossils in Avalonia come from the Charnian Supergroup of Leicestershire, UK [4,5,6,7,8,9,10,11,12,13,14] and the Conception and St. John’s groups across southeastern Newfoundland [15,16,17,18,19,20,21,22,23,24,25,26,27,28,29,30]. Both regions are characterized by volcaniclastic successions deposited in deep marine settings [31,32,33,34]—shallowing to fossiliferous offshore shelf and prodelta settings in Newfoundland [35]—and have fossil assemblages dominated by rangeomorph fossils [23,36,37].

**Figure 1:**
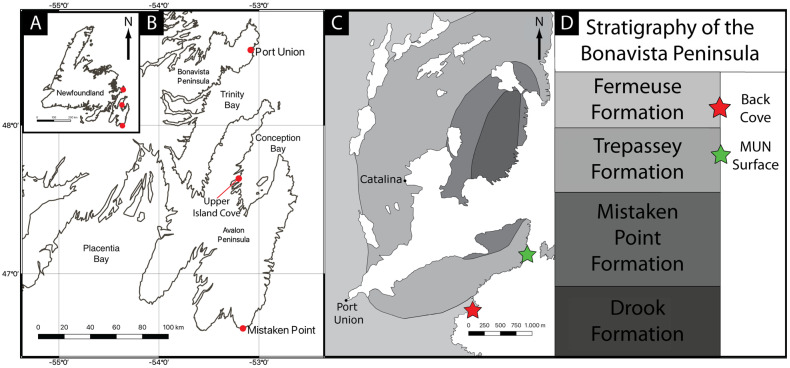
Location of the Catalina Dome on the Bonavista Peninsula of the island of Newfoundland, Canada, with key fossil sites highlighted with red circles (**A**,**B**) and the fossil sites with *Haootia* and *Mamsetia* highlighted by red and green stars, respectively (**C**,**D**). *Mamsetia* lies some 150 m stratigraphically below the type locality of *Haootia*.

The Ediacaran of Newfoundland consists of three distinct biotas: (1) those of the Mistaken Point type on the Avalon Peninsula [17,18,38,39]; (2) the exceptionally preserved Upper Island Cove assemblage [22,25,31,40]; and (3) the assemblages of the Catalina Dome on the Bonavista Peninsula [24,29,30,33,41,42,43] (Figure 1).

The shortage of geochronological data from the Bonavista Peninsula precludes reliable correlation with the now well-dated stratigraphy of the Avalon Peninsula assemblages [44,45], owing to the unreliability of lithostratigraphy as a correlative tool over hundreds of kilometres.

**Figure 2:**
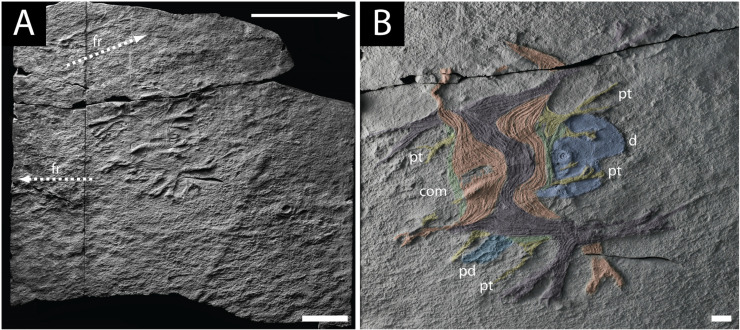
Holotype specimen of *Haootia quadriformis*. (**A**) image of the complete slab housed at The Rooms Provincial Museum, St. Johns, NL, with the inferred palaeocurrent indicated with a white arrow, scale bar 5 cm; (**B**) detail of the same specimen with groups of muscle fibres highlighted in colour: (pt) primary tentacle, (ped) peduncle, (com) coronal muscle, (d) disc; scale bar is 1 cm.

The holotype of the oldest known cnidarian, *Haootia quadriformis* (Figure 2A), comes from the Fermeuse Formation, at Back Cove, near Port Union on the Bonavista Peninsula [29,42] (Figure 1C,D). The type specimen was later removed from the field with permission of the Province of Newfoundland and Labrador, and is now housed in The Rooms in St. John’s Newfoundland (NFM F-994). The monotypic genus is based on plastotype OUM ÁT.424/p at the Oxford University Museum of Natural History. The well-preserved holotype and the paratype both include the preservation of muscle fibres, which have been used to interpret *Haootia* as a probable cnidarian [29,42,46,47].

Our re-examination of *Haootia* is based on the type material, including additional morphological and taphonomic considerations, as well as new, comparably preserved material attributed to a new genus and species of staurozoan cnidarian. We focus on the detailed description of the muscular tissue of the calyx and arms, and the relationship to the previously inferred basal disc [29,42]. The importance of *Haootia*—and our new genus *Mamsetia*—is emphasised by recent progress in understanding relationships within the Cnidaria [14,48,49].

## 2. Fossil Evidence for the Origins of the Cnidaria

The early fossil record of Cnidaria is largely that of the Medusozoa and Anthozoa, fossil examples which have been documented as present in a number of late Ediacaran sites [14,27,47,50]. Early interpretations of the frondose components of the Ediacaran macrobiotas typically compared most of the frondose taxa with pennatulacean anthozoans [51], and most discoidal taxa with medusozoans [52]. Those historical ideas have largely been replaced as many key taxa—particularly the Rangeomorpha and Arboreomorpha—are uniquely constructed and quite dissimilar to the Anthozoa [17,53]. Many of the Ediacaran fronds are composed of numerous self-similar modules in a fractal-like manner [7,10,19,20,22,25,26,37,54,55,56]. The discovery that many of the circular Ediacaran fossils originally interpreted as medusoids are either holdfast discs of frondose taxa (see reviews in [57,58,59], but also see [50]), or pseudofossils [27,60,61] has greatly decreased the number of inferred cnidarians in the Ediacaran.

Evidence for possible cnidarians from the late Ediacaran to Cambrian includes: (1) trace fossil evidence [27,62,63,64] of some un/lightly mineralized tubular taxa that have been compared to scyphopolyps (e.g., *Corumbella* [65]; and *Wutubus* [66]); (2) the conulariids *Vendoconularia* and *Paraconularia* [47,67,68,69,70], and the tetra-radial tubicolous medusozoan polyp *Auroralumina* [14]; and (3) the preservation of soft-bodied organisms in Cambrian lagerstätte [71,72]. In addition, many of the tubicolous taxa compared to the cnidaria are only known from the latest Ediacaran to Cambrian, making the cnidarian affinities of *Haootia quadriformis* all the more important to determine.

## 3. Taphonomy/Biostratinomy of *Haootia quadriformis*

Fossiliferous Ediacaran surfaces in the Catalina Dome (Bonavista Peninsula, Newfoundland) preserve exquisitely fine details of fossils in combinations of negative and positive epirelief [30], sometimes also involving a ferruginous veneer [43]. A combination of chemical and mechanical (salt) weathering affects the bedding surfaces by removing overlying ash layers and exposing the underlying fossils [73]. The main natural sources of damage to fossils consist of falling rocks, ice wedging, percussion impact of pebbles during storms, and scour from sea-ice [74].

**Figure 3:**
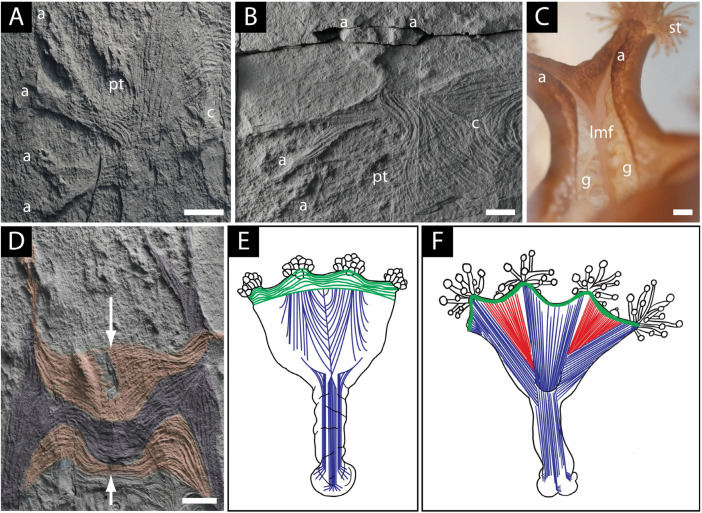
Comparative musculature of *Haootia quadriformis* and the modern staurozoan *Lucernaria*: (**A**) bundles of muscle fibres of branching arms (a) extending into the calyx (c) of the holotype of *H. quadriformis,* which is raised slightly above the bedding plane, scale bar 1 cm; (**B**) detail of the muscle fibres of the arms (a) and calyx (c) of *H. quadriformis*, showing their flattened nature and preservation slightly above the ambient bedding plane; note the twisting and branching of the arm, and also the muscles at the base of the primary tentacle (pt), scale bar 1 cm; (**C**) detail of an arm of *Lucernaria* showing the longitudinal muscle fibres (lmf) and branching of the arm (a) (partly covered by gonads (g)), branches of the arm are tipped by secondary tentacles (st), scale bar 0.25 cm; (**D**) groups of muscles of *H. quadriformis* showing the interlacing of one set of muscle fibres with the ends of the fibres from the opposite side of the calyx, forming an irregular vertical seam (arrowed) between the two groups of muscles (orange), and a second set of muscles (purple) meet the muscles of the opposing side of the calyx at its base, scale bar 1 cm; (**E**) musculature of the calyx of the extant staurozoan *Manania* showing the manner in which the longitudinal muscles of the arms (blue) meet to define the shape of the calyx in a manner similar to that seen in *Haootia* (after [91]); (**F**) musculature of the calyx of the extant staurozoan *Haliclystus*, showing the more open nature of the calyx with the longitudinal muscles of the arms (blue) not meeting at the margin of the calyx as inferred for *Mamsetia manunis* gen et sp. nov. (after [91])—perradial musculature (red) is not seen in *Mamsetia*. All scale bars 1 cm.

*Haootia* is distinctive among the Ediacaran fossils of Newfoundland in that it preserves muscle fibres (Figure 3A,B) [29,42]. The calyx of *Haootia* is preserved slightly above the surrounding bedding plane in a net-positive epirelief in the manner of some rangeomorphs (e.g., *Fractofusus* [56] and *Beothukis* [75]), although the fine details are generally negative-relief features on the (net) positive-relief fossil (Figure 3A,B,D).

Palaeocurrent data from frond orientations in the Catalina Dome show trends predominantly towards the southeast [24,76], which is consistent with independent current indicators [77]. The paradigm of frond “felling” has been challenged for some Ediacaran taxa [75], though some taxa—particularly some of the frondose arboreomorphs such as *Arborea spinosa* [78] and some rangeomorphs including *Avalofractus* and *Primocandelabrum*—do seem to have been erect during life [17,25,37,77,78,79]. Importantly, however, the erect fronds were likely oriented relative to clear-water currents, rather than density currents [77,79].

While sediment smothering by volcaniclastic material (either from a density current or ashfall settled from suspension [19,45,75]) is the most commonly invoked mode of preservation of the Ediacaran assemblages of Newfoundland, the preservation of *Haootia* was previously attributed to entrainment [42]. This would be comparable to the mode of preservation originally posited for the Upper Island Cove biota [25], but subsequently refuted [31,40]. Since the type material of *Haootia* is on a bedding plane, not within a bed, it was likely smothered in an obrution type event [80,81,82,83,84], not entrained within a sediment-laden current. The preservation of most of the arms on the inferred up-current side of the fossil (Figure 2B) supports the idea that the *Haootia* organism was affected by a unidirectional current prior to smothering. The calyx of *Haootia* should have forced the organism to be oriented down-current of a holdfast—if indeed one was present—but since this is not the case, we propose instead that the organism held onto the seafloor using the arms and tentacles on the up-current side of the calyx (see discussion of tentacle function in [85]). This is similar to the in-life position commonly assumed by modern staurozoans affected by currents [86]. The mode of preservation is thus most likely to have involved (tuffaceous) sediment obtrusion, and very early diagenesis [83].

The preserved morphology of *Haootia* is inferred as resulting from the preservation of muscle tissue, specifically bundles of muscle fibres [29]. Lineations within the musculature of *Haootia* are mostly concave epireliefs, implying that they represent collapsed muscle fibres (Figure 3A,B) rather than the permineralization or replacement of muscle tissue. Collapse of the muscle tissues must have happened after early lithification of the overlying ash, eventually allowing the underlying silt to fill the void left by the body [87,88]. The remobilization of silt likely happened only after degradation of the associated microbial mat during early burial [89]. Associated rangeomorph fossils on the MUN surface usually preserve the lower surface of the organism at or below the ambient bedding plane [30], which is apart from the normal positive relief preservation of some stems [90].

The muscles of *Haootia* constitute a four-sided calyx with pairs of arms at each corner (Figure 2B). Where groups of muscle fibres cross one another, it is the uppermost set of muscle fibres that is preserved, though often it is possible to determine the position of underlying muscle fibres where they can deform overlying muscle layers (Figure 3A,B). Tissues that lay below the uppermost musculature of the calyx of *Haootia* were not cast by the overlying tuff and were therefore not preserved. The preservation of muscle fibres is relatively common in chordates [92,93], and in some early invertebrates such as Cambrian lobopods [94,95] and Cnidaria [50]. With taphonomic limitations in mind, it is nonetheless clear that the preservational quality of the holotype of *Haootia* is truly exceptional [29,42].

## 4. Descriptive Palaeontology

Careful photography and tracing of groups of inferred muscle fibres that form the basis of the redescription of the type material of *Haootia* herein support its inclusion in the Cnidaria [29,42,46], and invite comparisons with the tetra-radial bauplan of the Staurozoa as detailed below.

### 4.1. Longitudinal Muscles of the Calyx and Arms of H. quadriformis

The predominant feature of the *Haootia quadriformis* holotype is the almost-square outline of the calyx [29]. The calyx is composed of eight sets of longitudinal muscles, all of which extend beyond the calyx into an arm of the fossil (Figure 2B). The more completely preserved of the arms bifurcate close to their tips (Figure 2B). Preserved morphology of the tips of the arms is completely lacking in the holotype, suggesting that the tissues were rapidly degraded, and thus difficult to fossilize.

The arms of modern stauromedusae have clusters of secondary tentacles at their tips (cf. Figure 3C) that are used for grasping food particles, including small invertebrates [96]. In the laboratory, the secondary tentacles of the arms of *Lucernaria* are commonly seen to brush the sediment surface (Supplementary Information S1), which suggests possible surficial deposit feeding. The arms of Staurozoa are also important in some species for locomotion on both hard and soft substrates, usually involving initial attachment using the secondary tentacles (at the ends of the arms) and (primary tentacle) anchors, followed by contraction of the longitudinal muscles of the arms [97] (Supplementary Information S2).

While no tentacular structures are preserved at the ends of the arms of *Haootia*, it is possible, given the gross morphological similarity with the Staurozoa, that there were clusters of secondary tentacles at the tips of at least some of the arms. The preservation potential of delicate cnidarian tentacles is extremely low. Even in konservat lagerstätte, in which muscle tissues are moderately well preserved, fine tentacles are generally poorly preserved [59,98,99]. More robust cnidarian tentacular structures have recently been inferred from the Ediacaran *Auroralumina* ([14] though no muscle preserved) and the Cambrian *Burgessomedusa* [100].

The corners of the calyx in the holotype of *Haootia* are characterized by pairs of arms (Figure 2B). The musculature of one set of arms—one of which is present in each corner of the calyx—is arranged such that it meets the musculature of longitudinal muscles from the opposite side of the calyx at its base (Figure 2B, coloured purple). The other set of arms have fibres that are more curved in the calyx portion, and connect to the equivalent muscle fibres of the adjacent paired arms (Figure 2B and Figure 3D, coloured orange), as in some modern staurozoans (Figure 3E).

**Figure 4:**
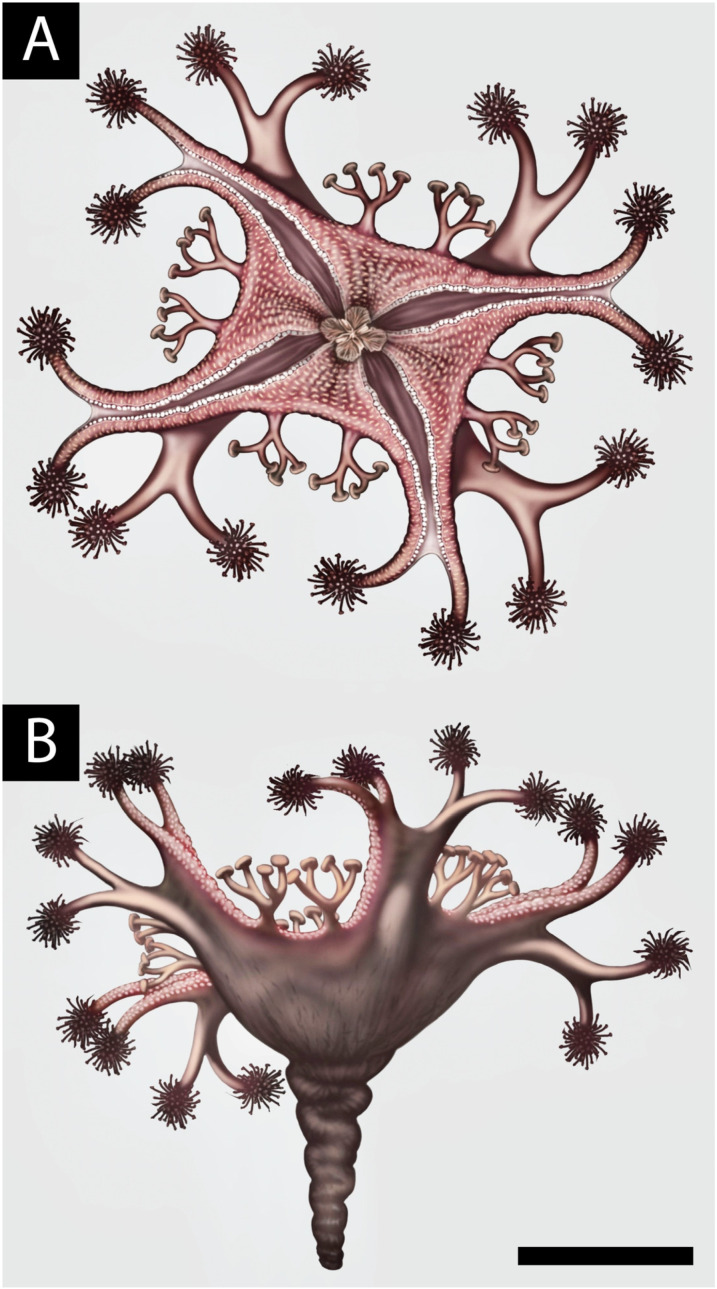
The holotype of *H. quadriformis* reconstructed by analogy with modern stauromedusae, showing the paired arms at each corner of the calyx tipped with hypothetical secondary tentacles and shorter divided primary tentacles at the margin of the calyx with hypothetical adhesive pads inferred from the morphology of modern stauromedusae. Approximate scale bar 5 cm. (**A**) is an oral view and (**B**) is the lateral view of the calyx and peduncle © Bob Nicholls.

In absence of preserved tissues from the oral surface of the arms (e.g., gonads and gastric tissues, which would be highly labile and have low fossilization potential, Figure 3C), it is possible that some of the arms were specialized for locomotion rather than feeding (Figure 4).

We note that a free-living developmental stage of the parasitic cnidarian *Polypodium* [101,102] has four walking tentacles and four feeding tentacles, all with longitudinal muscles [102,103]—an architecture somewhat comparable to *Haootia*. *Polypodium* might, therefore, be a good supplementary biomechanical model to understand the eight-armed *Haootia*. No taxonomic affinity is inferred, however, as *Polypodium* is probably a highly derived cnidarian.

### 4.2. Marginal Muscles of the Calyx of H. quadriformis

**Figure 5:**
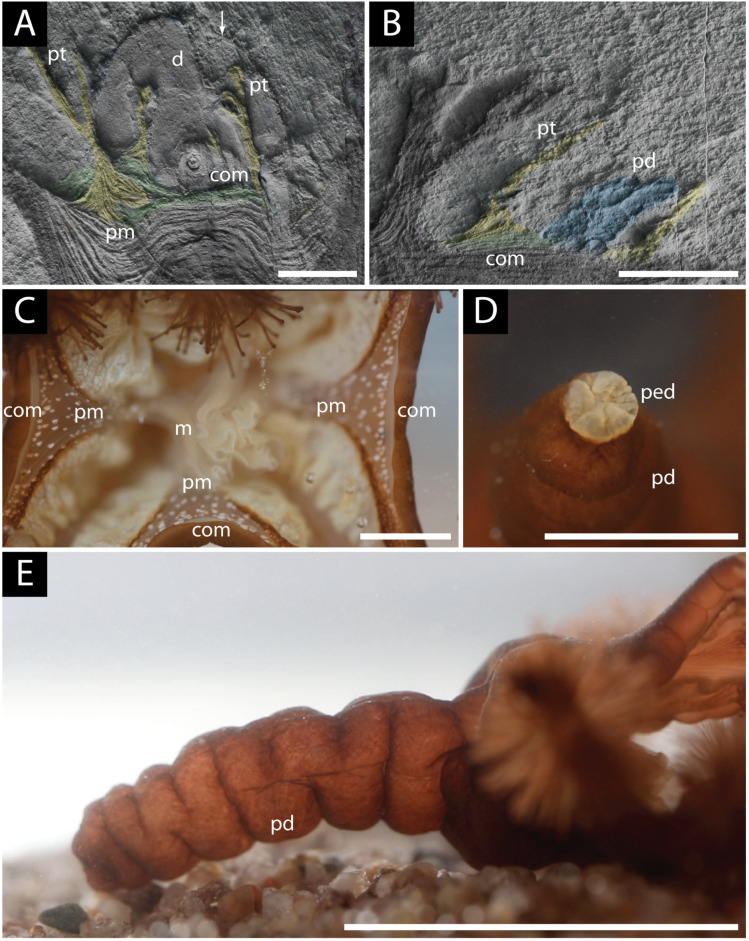
Comparative morphology of *Haootia* and a modern staurozoan (*Lucernaria*): (**A**) primary tentacles (pt) with triangular per-radial musculature (pr) showing their filiform morphology and association with the coronal muscle (com), scale 1 cm; also note the lifting of the margin of a disc (d) by one of the filiform primary tentacles (arrow); (**B**) detail showing the inferred peduncle of *Haootia* (pd) adjacent to a primary tentacle (pt) with coronal muscle (com) (scale 2 cm); (**C**) coronal muscles (com) and per-radial muscles (pm) of modern *Lucernaria* and the central manubrium (m), scale 1 cm; (**D**) pedal disc (ped) of *Lucernaria*, which is small and comparable in width to the peduncle (pd), scale 5 mm; (**E**) morphology of the peduncle (pd) of *Lucernaria*, approx. scale 3 cm.

Our re-examination of the holotype *Haootia* has demonstrated the presence of low-relief bands of narrow muscle fibres that lie parallel to the margins of the inferred calyx (Figure 5A,B), which are consistent with *Haootia* having a stauromedusan-like calyx [91]. The coronal (marginal) muscles of Staurozoa form a similar narrow band on the margin of the calyx (Figure 5C) and serve the function of closing it, for example during times of environmental stress [91] or to decrease the aspect ratio of the calyx in a strong current [96]. The coronal muscles of *Haootia* do not continue across the arms, suggesting that they were either discontinuous, as they are in many staurozoans, or that they ran inside the arms of the holotype and are thus not preserved because of preferential casting of the overlying arms.

The poorly developed marginal muscles of the Staurozoa are in marked contrast with those of free-swimming medusozoans (e.g., Scyphozoa and Cubozoa), in which contractions of the marginal/circular muscles are used for propulsion [104]. The paucity of marginal musculature in stauromedusae is a function of their sessile epibenthic mode of life [105]. It has previously been stated that the coronal musculature of *Haootia quadriformis* was extremely well developed, and more comparable to that of pelagic cnidarians [46]. Those assertions are based on misinterpretation of some of the longitudinal muscles of the arms that lie parallel to the margin of the calyx circular/coronal muscle tissue. That same misinterpretation of some of the longitudinal musculature of *Haootia* as coronal has previously cast some doubt on the interpretation of *Haootia* as a crown group cnidarian [14,46,69].

### 4.3. Marginal Tentacles of H. quadriformis

The holotype of *Haootia* has short tentacles associated with the margin of the calyx [29] (Figure 2B and Figure 5A,B). The presence of “small branching structures” was noted in the diagnosis of *Haootia* [29], but they were not recognized as being analogous to the primary tentacles of Staurozoa as they are herein. Among the Cnidaria, similarly positioned tentacles are common in stauropolyps, but in the stauromedusae these primary tentacles may be: (1) resorbed [106]; (2) metamorphosed into inter-radial anchors [107]; (3) modified in situ at their juvenile position [106]; or (4) become capitate and migrate into clusters with the secondary tentacles at the end of the arms [107]. When stauromedusan primary tentacles are located on the calyx margin, they are intimately associated with the coronal muscle, and are controlled by perradial musculature [91]. The marginal tentacles on the coronal/marginal muscles of the calyx of *Haootia* all have well-developed musculature (Figure 2A). The distal ends of the marginal tentacles appear to be filiform, although the preservation is only of the internal musculature, not of the gross morphology of the original structure (Figure 5A).

Modern stauromedusae do not generally have pairs of perradial primary tentacles, although many stauropolyps have eight primary tentacles in the early phases of growth [108], four of which are either resorbed or migrate to the ends of the arms [107]. Rare examples of *Halyclistus* have been documented with paired perradial anchors (e.g., [106], their Figure 7j), although whether this phenotype is associated with genetic or environmental drivers is unclear (see [109]).

Among modern Staurozoa, the amyostaurid *Calvadosia* has long, prominent, primary tentacles, similar to those of *Haootia*, with a distinctive V-shaped region of perradial muscle that extends beyond the marginal muscle in a manner that is comparable to the musculature at the base of the marginal tentacles in *Haootia* (Figure 5A). The long primary tentacles of *Calvadosia* are primarily used to modify the position of the calyx by attaching the calyx to the substrate (e.g., during locomotion [97]). In the holotype of *Haootia*, it seems that one of the marginal tentacles has lifted a portion of the margin of a large holdfast structure, revealing the sub-disc sedimentary texture underneath, suggesting a similar grasping function and the facility to contract the muscles therein (arrowed on Figure 5A).

### 4.4. Peduncle and Basal Attachment of H. quadriformis

**Figure 6:**
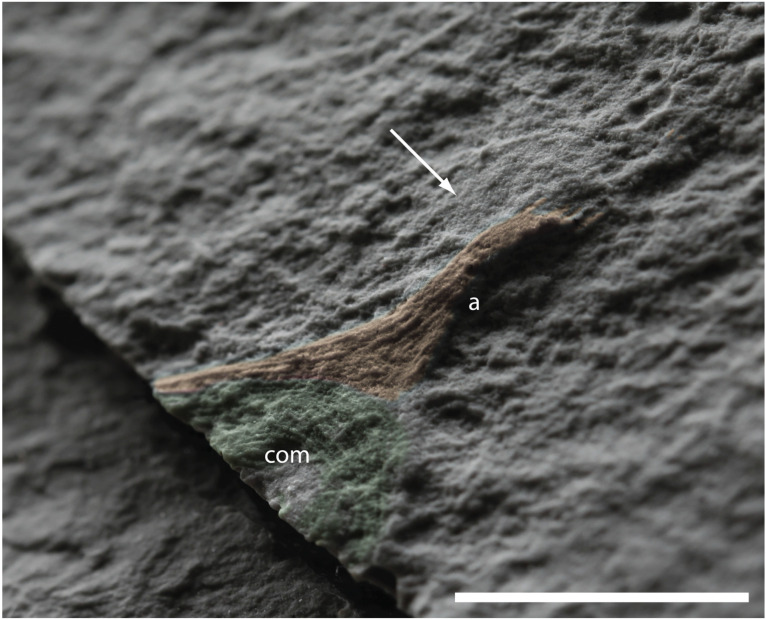
Detail of the specimen formerly designated as the paratype of *H. quadriformis* by [29] and compared to *Mamsetia manunis* gen. et sp. nov. herein: longitudinal muscle of the arm (a) is highlighted in orange—note that there is only one arm at the corner of the calyx, not 2 as in the holotype of *H. quadriformis*. The preserved portion of circular/coronal muscle (com) of the oral surface is more complete than in stauromedusae, but comparable to the oral surface of stauropolyps. Note the smooth area above the arm (arrowed), which was interpreted as a basal disc previously. Scale bar 1 cm.

In the description of the holotype [29], the calyx and arms of *Haootia* were borne atop a short stalk or peduncle, which was attached to a large, rather smooth disc (Figure 2B). The paratype of *Haootia* (Figure 6) was also considered to be a partial specimen of a stalk/peduncle and associated disc.

Immediately adjacent to the calyx of the holotype is a short, roughly conical, wrinkled structure that has not been discussed in previous studies (Figure 5B). The wrinkled structure is quite unlike any of the described Ediacaran taxa from the Catalina Dome [24] or other Ediacaran deposits in the Newfoundland sections [23,36,37]. The peduncle of many modern staurozoans does, however, have a similar wrinkled outer surface, and it is to this structure that this fossilized tissue is compared (Figure 5E).

The peduncle of many, but not all [91], modern Staurozoa contain longitudinal musculature that allows its contraction, either to retract the calyx towards the substrate or to allow locomotion [98,106]. Modern Staurozoa are generally considered to locomote by alternate adhesion of the arms and peduncle to the substrate, combined with contraction of longitudinal muscles in both structures to produce a tumbling or inchworm-like motion [86]. We note, however, that in laboratory settings, the modern staurozoan *Lucernaria* predominantly locomotes on flat, sandy substrates with the manubrium down, using the arms and adhesive properties of the secondary tentacles at their tips (Supplementary Information S1).

There is no evidence for longitudinal muscles being preserved in the peduncle-like structure of *Haootia*. The peduncle might have also contained muscles, but not as the outermost tissue preserved.

We have not found compelling evidence of any direct biological association between the holotype and a discoidal holdfast-like structure—the affinities of the paratype are discussed below. The disc associated with the holotype of *Haootia* is much larger than the pedal disc of modern Staurozoa (Figure 5D), which use the pedal disc for temporary attachment. All known stauromedusae are motile, a mode of life that would be incompatible with a pedal disc that is half the width of the calyx and approximately ten times wider than the peduncle itself [46]. In the absence of reliable evidence linking *Haootia* with a holdfast-like disc, it becomes pertinent to consider a mobile epibenthic mode of life.

### 4.5. Issues with the Paratype of Haootia quadriformis

The partial specimen described as the paratype of *Haootia* [29] is a field photograph of a small partial specimen of just over 2 cm in length from the MUN surface [42]. It has been considered to have an associated disc of very low relief that is approximately 5 mm in diameter (Figure 1f in [29]; Figure 6). The newly accessioned cast of the former paratype of *Haootia* (NFM F-3976) does not show clear evidence of a disc, just a smooth area of adherent tuffite (Figure 6, arrowed).

Additionally, the arrangement of muscle fibres in what has previously been considered a peduncle most closely resembles the longitudinal muscles of the arms of the holotype. The musculature of the calyx at the basal end of the preserved longitudinal muscle is similar to the coronal musculature expected at the margin of a staurozoan-like calyx (Figure 5C). It seems most parsimonious, therefore, to consider that the specimen preserves the circular marginal muscles of the calyx and the longitudinal muscles of one arm rather than a peduncle and part of the calyx. The absence of two arms at the corner of the calyx—the case in the holotype of *H. quadriformis*—is, however, problematic to its inclusion in the genus *Haootia*.

The upper surface of the calyx in the former paratype of *Haootia* is not open, like those of stauromedusae, but is covered by concentric muscles, which are morphologically comparable to the musculature of stauropolyps and also Hydrozoa [104]. We hope to find further specimens of small, *Haootia*-like fossils, but given the presence of just one (unpaired) arm, and the fact that the calyx is closed, it seems unlikely that the specimen belongs in *Haootia*. It could, perhaps, represent an early developmental stage of an undescribed staurozoan or hydrozoan.

### 4.6. New Fossils with Muscular Preservation from the MUN Surface

**Figure 7:**
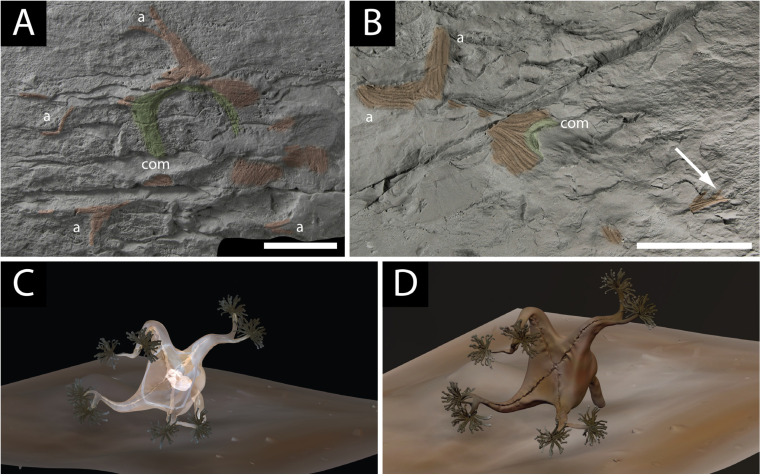
Fossil material and reconstruction of *Mamsetia manunis* gen. et sp. nov.: (**A**) holotype of *M. manunis* preserving a thick band of coronal muscle ((com) coloured green and has no primary tentacles) and longitudinal muscles of the arms ((a) coloured orange)—note that the calyx is not coherent as it is in *H. quadriformis*, and the specimen was damaged by rockfall in the field, scale bar 2.5 cm; (**B**) large specimen of *M. manunis* (scale bar 5 cm), orange longitudinal muscles of the arms, scale bar 5 cm (a), and possible green coronal muscle (com)—note the branching at the tips of the arms (arrowed); (**C**) reconstruction of *Mamsetia manunis* showing the four arms and a staurozoan body plan with no primary tentacles. The exterior tissue of the calyx is made transparent to highlight the positions of the internal coronal and longitudinal musculature; (**D**) reconstruction of the mode of life of *Mamsetia manunis*.

We describe herein for the first time several partial specimens of a large four-armed tetra-radial cnidarian-like muscular organism (Figure 7A–D) from the MUN Surface in the Trepassey Formation of the Catalina Dome. Like the specimen that was originally designated as the paratype of *Haootia quadriformis*, all of the new specimens have only one arm on each corner of the calyx, the state in all modern Staurozoa. The new material has a band of well-developed coronal musculature that lies inside the arms (Figure 7A) and includes some very large specimens (Figure 7B,C), all of which are elongated in the direction of the inferred palaeocurrent on the MUN Surface [76]. None of the new specimens is completely preserved; the most commonly preserved tissues are the coronal muscles and the longitudinal muscles of the arms. The tissues of the arms and calyx appear to be less well connected to one another than in the holotype of *H. quadriformis*, perhaps due to the partial decay of connective tissues or original morphology (cf. Figure 3F). In some species of modern staurozoan, the musculature of the arms does not meet that of adjacent arms to form a muscular margin to the calyx, but instead all muscles meet at the base of the calyx [91] (Figure 3F). The calyx is, thus, not defined by the musculature of the arms, so the arms are less likely to remain associated with one another post-mortem once the calyx and other soft tissue decays. The specimens are preserved as positive relief structures, and have thus been subjected to abrasion by rockfall onto the surface, which degrades the specimens. The four arms divide close to their tips in the best-preserved specimens (Figure 7A). The large size of the specimens suggest that the number of arms present is a characteristic of adult organisms, and the arrangement of the musculature of the arms (one in each corner) similarly suggests that the small, former paratype of *H. quadriformis* should, instead, be considered conspecific with this new material (on the same surface), not *Haootia*, as described below.

## 5. Systematic Palaeontology

Phylum: Cnidaria Hatschek 1888

Subphylum: Medusozoa Peterson 1979

Class: Staurozoa Marques and Collins 2004

Genus: *Haootia* Liu et al., 2014

Diagnosis: per species.

Type Species: *quadriformis* by monotypy

Holotype: NFM F-994 (The Rooms Provincial Museum, St. John’s, NL, Canada); plastotype OUM ÁT.424/p (Oxford University Museum of Natural History, UK)

2014 *Haootia quadriformis* Liu et al., 2014, figures 1a–e and 2a,

non v2014 *Haootia quadriformis* Liu et al., 2014, figure 1f [moved to *Mamsetia manunis* gen. et sp. nov.]

Emended diagnosis: soft-bodied, tetra-radially-symmetrical fossil with a calyx comprised of numerous long linear fibres that extend into eight arms. The arms divide dichotomously to form smaller sub-branches. The margin of the calyx is marked by a band of circular muscle. The short pedicle structure is not attached to a substantive holdfast or disc. The margin of the calyx has at least two short narrow branches which have muscular tissue at their centre and which divide distally.

Description: the calyx is formed of muscular tissues, as is the case in some modern Staurozoa (e.g., *Manania*, see [91], Figure 3E), with the musculature of adjacent arms meeting to form the margin of the calyx between the arms. The short branches on the margin of the calyx are considered to be staurozoan-like primary tentacles and are preserved in the form of their internal musculature. The contractile nature of the tissue is evidenced by the lifting of an adjacent holdfast disc, possibly post-mortem. The pedicle does not preserve any muscle.

Remarks: the previously inferred disc ([29]) is considered to be an accidental association herein. The type description included the designation of a paratype, which is a very small partial specimen that we consider as having only one arm preserved along with part of the coronal/marginal muscle. The former paratype comes from the same surface as the newly described *Mamsetia manunis* gen. et sp. nov., which similarly has only one arm at each corner of the calyx, and is thus tentatively transferred to that genus herein. While *H. quadriformis* has eight rather than four arms, as is normal in modern Staurozoa, it has sufficient other morphological characters (tetra-radial, peduncle, marginal tentacles) and muscular architecture to include it in that class (Supplementary Information S2).

Genus: *Mamsetia* gen. nov.

v. cf. *Haootia quadriformis* Liu et al., 2014, figure 1f [paratype of *H. quadriformis* Liu et al., 2014].

Diagnosis: per species.

Etymology: *Mamsetia* is derived from “Mamset”, which is a word in Beothuk (the language of the indigenous people of Newfoundland at the time of European colonization) meaning ‘living’.

Type species: *manunis* sp. nov. by monotypy.

Etymology: The species name *manunis* is derived from “Manune or Manume”, which is Beothuk for pitcher/cup, pertaining to the shape of the calyx.

Type Locality: MUN Surface in the Port Union Member of the Trepassey Formation near Green Island, Port Union, Bonavista Peninsula, NL (Figure 1C,D).

Holotype: NFM F-4011 (Figure 7A).

Paratype: NFM F-4012 (Figure 7B).

Other material: NFM F-4013; NFM F-4014; NFM F-4015; NFM F-4016; and possibly NFM F-3976 [former paratype of *Haootia quadriformis*].

Diagnosis: A tetra-radial soft bodied organism comprised of preserved muscle tissue, defining the four corners of the calyx. The arms bifurcate distally into two equal muscular structures. Coronal musculature broad and well-developed, without marginal tentacles. No basal attachment disc is present.

Description: the manner in which the muscles of the arms join to form the calyx is not documented in any of the specimens. The well-preserved coronal muscles of the holotype do not have any evidence of structures comparable to the primary tentacles of some Staurozoa, e.g., *Haliclystus* [91] (Figure 3F), distinguishing it from *H. quadriformis*. None of the specimens of this species shows evidence of a prominent basal disc, as previously invoked for *H. quadriformis* [42].

Remarks: the four arms of *M. manunis* support the attribution of the genus to the Staurozoa, and indirectly support claims that *Haootia* was also a staurozoan. While the beds with both *H. quadriformis* and *M. manunis* have not yet been dated precisely, the best lithostratigraphic estimate of around 565 Ma [42] would make these the oldest crown group Cnidaria currently known, with *M. manunis* lying in a bed 150 m stratigraphically below that from which *H. quadriformis* was described. In gross morphology at least, *Mamsetia* is considered to have looked much like a modern staurozoan (Figure 7C,D; Supplementary Information S2), with musculature that does not encapsulate the whole calyx but forms bands within it (Figure 7C; compare Figure 3F).

## 6. Discussion

**Figure 8:**
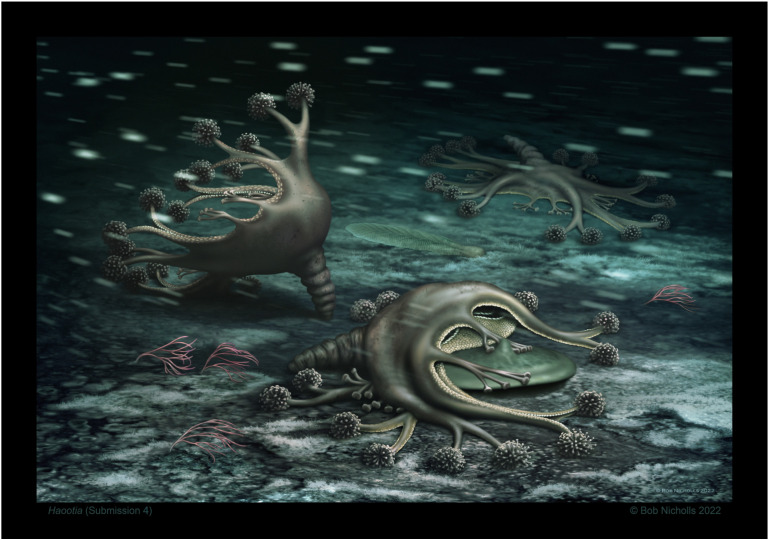
Artist’s reconstruction of the seafloor with several *Haootia quadriformis* reconstructed as motile cnidarians with eight arms, marginal tentacles, and a peduncle. The *Haootia* are living in a current (left to right) on a microbe-dominated seafloor alongside a reclining *Charnia* sp. and possible alga *Parviscopa bonavistensis*. Note that one of the muscular primary tentacles of the foreground *Haootia* is lifting the margin of a discoidal holdfast-like form, as in the holotype specimen © Robert Nicholls.

*Mamsetia manunis* and *Haootia quadriformis* have several characters typical of stauromedusae, namely: (1) a tetra-radial calyx defined by a total of either four or eight arms, located on the corners of the calyx; (2) the open end of the calyx has a narrow marginal band of coronal/marginal muscle; (3) in *H. quadriformis*, two marginal tentacles are present on each margin of the calyx, each with associated musculature; (4) the arms of *Haootia* have musculature that defines the shape and symmetry of the calyx, much like that of the modern staurozoan *Manania*, whereas that of *Mamsetia* gen. nov. is closer to that of *Haliclystus* [91]; (5) *Haootia quadriformis* preserves a flexible peduncle without permanent attachment to the substrate; and (6) contrary to earlier reports [29], there is no good evidence to support the presence of a broad holdfast disc homologous to the pedal disc of modern staurozoans (Figure 8).

During staurozoan metamorphosis from stauropolyp to stauromedusa, four of the eight primary tentacles present in stauropolyps have a variety of fates: they may be retained into adulthood, while some are resorbed or transformed into either rhopalioids or rhopalia [109,110]. *Haootia quadriformis* retained at least eight marginal tentacles into adulthood (Figure 2A), and *Mamsetia manunis* had none preserved.

*Haootia quadriformis* (Figure 2) differs from modern stauromedusae in having eight arms that define the tetra-radial symmetry—rather than the four of *Mamsetia* (Figure 7)—but it otherwise closely resembles modern staurozoa. The presence of four additional arms in modern staurozoan polyps opens the possibility of interpreting *H. quadriformis* as an early or “crown” staurozoan, which might have retained the eight arms as a plesiomorphic trait. The preserved features of the newly described *M. manunis* are all present in modern staurozoa, thus supporting the presence of Ediacaran staurozoan cnidarians. The preservational style of *Haootia* and *Mamsetia* is dependent on the casting of muscle tissues [29]. The arms and primary tentacles of *Haootia* only preserve the muscle tissues, with no clear evidence for terminal tentacular structures. If *Haootia* was, indeed, a member of the Cnidaria, it is likely to have either borne nematocysts or adhesive tentacle clusters to aid feeding and/or locomotion (Figure 8).

The subumbrellar surface of the arms in modern Staurozoa commonly bear gonads, but since the delicate structures of the subumbrellar surface (gastrovascular system, manubrium, etc.) of *Haootia* and *Mamsetia* are not preserved, it remains possible that: (1) either all arms were gonad-bearing, like modern stauromedusae (Figure 4); (2) that one set of arms in *H. quadriformis* was purely designed for locomotion (Figure 4); or (3) if all the arms of *Haootia* and *Mamsetia* had no such gastrovascular system, the organism would be less Staurozoa-like than reconstructed herein, but given the tetra-radial symmetry it would likely still be considered a cnidarian. The preserved anatomy of *Haootia* demonstrates that it was a benthic cnidarian of medusozoan affinity, with longitudinal muscles within the calyx and weakly developed marginal/coronal muscles around the mouth of the calyx.

Preliminary phylogenetic analyses performed on a pre-existing morphological dataset of modern and extinct cnidaria [14] consistently retrieved *Haootia* and *Mamsetia* as derived (crown-group) staurozoans (Supplementary Information S2).

## 7. Palaeobiological Implications

The depositional setting of the Ediacaran Conception Group in the Bonavista region of Newfoundland is that of a distal shelf or slope, which is dominated by turbidites and debrites [33,34,35]. The palaeoenvironment of deposition is compatible with the wide range of environments from which modern staurozoans are reported [111,112]. The palaeobiology and mode of life of many elements of the Ediacaran biota remain contentious, including: the harbouring of chemosymbionts [113], phagotrophy [113,114], osmotrophy [115,116], suspension feeding using specialized organs [23,51], whole-body suspension feeding [117], and/or gas exchange [117,118]. If we accept *Haootia* and *Mamsetia* as staurozoan cnidarians, they are likely to have had a primarily heterotrophic mode of life, either through the active predation on plankton or feeding on detrital material on the seafloor, by analogy with modern staurozoa [106,119,120,121].

The preserved muscles of both the arms and primary tentacles of *Haootia* are consistent with active locomotion and with our assertion that it did not, in fact, have a large basal anchoring disk, contra [29,46]. If *Haootia* and *Mamsetia* are accepted as stauromedusae, a small pedal disc was likely present at the end of the peduncle.

## 8. Phylogenetic Implications

It is commonly agreed that the Metazoa and Eumetazoa evolved in either the Cryogenian or the Tonian [122,123], an estimate that significantly predates their first appearance in the Ediacaran fossil record. The clearest evidence of an Ediacaran animal in the oldest known Avalon Assemblage biotas is in the form of trace fossils [27,63,64] and the medusozoan body fossil genera *Haootia, Mamsetia*, and *Auroralumina* [14,29]. Metazoan affinities have also been proposed for: *Thectardis* [124], the Arboreomorpha [125], and the Rangeomorpha [12].

Traditionally, the Porifera have been considered a monophyletic group, diverging from other animals as early as in the Cryogenian [126,127], but this has been challenged by some recent studies, which see Porifera as a paraphyletic group in the metazoan stem instead [114,128]. The paraphyly hypothesis indirectly supports Cavalier-Smith’s [129] hypothesis that the eumetazoans evolved from a pre-sponge-grade ancestor. Some studies propose the Ctenophora as the earliest group of animals [130,131,132,133], with sponges evolving later with the other animal groups in the late Ediacaran. This second hypothesis has been proposed as evidence to explain the apparent absence of sponges from the rich Ediacaran fossil record [134], but see the *Protospongia*-like *Helicolocellus cantori* [135]. The presence of staurozoan cnidarians in the Ediacaran fossil record does, however, require the presence of one or both of their two possible sister groups, the Porifera and the Ctenophora, even though their phylogenetic positions relative to the Metazoa are still under debate [130,131,136]. The presence of the genera *Haootia* and *Mamsetia* in the Ediacaran thus has important implications for the understanding of early animal evolution and the calibration of molecular clocks, but also for the evolutionary timing and phylogeny of the Cnidaria [137].

Staurozoans are traditionally considered to be an early-diverging monophyletic group within the subphylum Medusozoa, an interpretation which has been largely confirmed by molecular data [69,138], though the phylogenetic relationships within the Medusozoa are still debated [49,139].

Since the Anthozoa is a sister group to the Medusozoa, anthozoans are expected in the Ediacaran, with the inferred divergence date for the two groups thought to be during the Tonian [140]. The absence of confirmed anthozoan fossils from the Ediacaran can be explained by the late evolution of skeletal structures and the simple external morphology of the Actinaria [64].

If staurozoans are accepted as primitive medusozoans, they would play an important role in the debate over whether a polyp or a medusa was the plesiomorphic trait of the Cnidaria (and therefore the Medusozoa). It has been suggested that the Medusozoa might have evolved from a polyp-like ancestor, with the absence of a medusoid stage in the Anthozoa being the ancestral state rather than the result of later evolutionary loss [141], however, the stauromedusae of *Haootia* and *Mamsetia* are large and have well-developed muscular structures in the arms.

## 9. Conclusions

*Haootia quadriformis* is one of the most iconic of Ediacaran taxa and the earliest example of a staurozoan cnidarian in the fossil record, to which we now add a second genus, *Mamsetia manunis*. The exceptional preservation of muscle tissues in both genera allows the recognition of a tetra-radial symmetry that supports their inclusion in the Cnidaria. This work rejects the hypothesis that *Haootia* was attached to the seafloor by a large holdfast disc, and instead identifies a staurozoa-like peduncle. The suggestion that *Haootia* had very highly developed marginal muscles unlike those of extant stauromedusae [106] was based on misinterpretation of the distribution of the longitudinal muscles associated with the arms of the holotype, and was rejected herein following the recognition of delicate marginal/coronal musculature similar to that of modern stauromedusae. The marginal branches of *Haootia quadriformis* were here reinterpreted as being analogous to the perradial primary tentacles that lie at the margin of the calyx of modern Staurozoa. Primary tentacles are, as yet, unknown in *Mamsetia manunis*.

*Haootia quadriformis* differs from modern Staurozoa in having eight arms and at least two perradial marginal tentacles associated with the coronal muscles of each side of the calyx, traits which could be considered plesiomorphic to the class. *Mamsetia manunis* has the conventional four arms of the Staurozoa, but with no perradial musculature or primary tentacles preserved. We therefore consider that *Haootia* and *Mamsetia* are the best-preserved candidates for being the earliest medusozoans, and that they were morphologically similar to modern Staurozoa. The presence of cnidarians in the Ediacaran implies that Ctenophora and Porifera had already evolved during the Neoproterozoic, even though they might have been excluded from the peculiar Ediacaran taphonomic windows. The former paratype of *Haootia* might also conceivably be an early developmental (cf. stauropolyp) stage of either *Mamsetia* or *Haootia*, but further consideration requires the identification of more small specimens in the field.

## Data Availability

Data are contained within the article and Supplementary Materials.

## Supplementary Materials

The following supporting information can be downloaded at: https://www.mdpi.com/article/10.3390/life14091096/s1, Supplementary Information S1: locomotion of *Lucernaria* sp.; and Supplementary Information S2: cladistical analysis encompassing *Haootia* and *Mamsetia.*
