# Supplementary material for: The Palaeobiology of Two Crown Group Cnidarians: Haootia quadriformis and Mamsetia manunis gen. et sp. nov. from the Ediacaran of Newfoundland, Canada
Source: Life (Basel). 2024 Aug 30;14(9):1096. doi: 10.3390/life14091096 (PMC11432848; doi:10.3390/life14091096)
Supplement: Supplementary file 1 [file life-14-01096-s001.zip › Supplementary material S2.docx]

Supplementary material:

Cladogram based on a dataset compiled by Dunn et al. (2022), incorporating pre-existing datasets (Duan et al., 2017; Ou et al. 2017; Zhao et al., 2019). The dataset was trimmed to retain 42 informative species and 104 morphological traits. Analyses were performed in RStudio 4.0 using the phylogenetic package “Phangorn” (Schiliep et al., 2011).

A maximum parsimony methodology (Nixon, 1999) produced 719 rooted trees, which were then combined to inform a consensus-tree using the R package Ape 5.0 (Paradis et al., 2019).


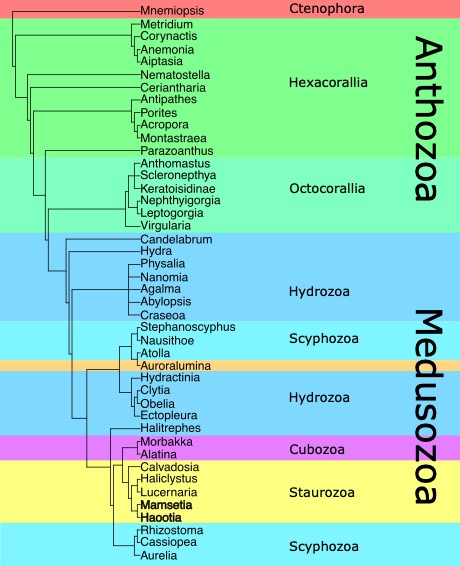


Baichuan, D.; Dong, X.; Porras, L.; Vargas, K.; Cunningham, J.A.; Donoghue, P.C.J. The early Cambrian fossil embryo *Pseudooides* is a direct-developing cnidarian, not an early ecdysozoan. *Proceedings of the Royal Society B: Biological Sciences* **2017,** 284, 20172188. doi:[10.1098/rspb.2017.2188](https://doi.org/10.1098/rspb.2017.2188).

Qiang, O.; Han, J.; Zhang, Z.; Shu, D.; Sun, G.; Mayer, G. Three Cambrian fossils assembled into an extinct body plan of cnidarian affinity.” *Proceedings of the National Academy of Sciences* **2017** 114, 8835–40. doi:[10.1073/pnas.1701650114](https://doi.org/10.1073/pnas.1701650114).

Nixon, K. The parsimony ratchet, a new method for rapid parsimony analysis. *Cladistics* **1999**, 15, 407–14.

Emmanuel, P.; Schliep. K.. “Ape 5.0: An environment for modern phylogenetics and evolutionary analyses in r.” *Bioinformatics* **2019** 35, 526–28. <https://doi.org/10.1093/bioinformatics/bty633>.

Yang, Z.; Vinther, J.; Parry, L.A.; Wei, F.; Green, E.; Pisani, D.; Hou, X.; Edgecombe, G.D.; Cong, P. Cambrian sessile, suspension feeding stem-group ctenophores and evolution of the comb jelly body plan.” *Current Biology* **2019**, 29, 1112-1125.e2. doi:[10.1016/j.cub.2019.02.036](https://doi.org/10.1016/j.cub.2019.02.036).
